# Is it time to turn our attention toward central mechanisms for post-exertional recovery strategies and performance?

**DOI:** 10.3389/fphys.2015.00079

**Published:** 2015-03-17

**Authors:** Ben Rattray, Christos Argus, Kristy Martin, Joseph Northey, Matthew Driller

**Affiliations:** ^1^Discipline of Sport and Exercise Science, Faculty of Health, University of CanberraCanberra, ACT, Australia; ^2^University of Canberra Research Institute for Sport and Exercise, University of CanberraCanberra, ACT, Australia; ^3^Department of Sport and Leisure Studies, The University of WaikatoHamilton, New Zealand

**Keywords:** recovery, brain, mental fatigue, sleep, nutrition

## Abstract

**Key Points**

- Central fatigue is accepted as a contributor to overall athletic performance, yet little research directly investigates post-exercise recovery strategies targeting the brain
- Current post-exercise recovery strategies likely impact on the brain through a range of mechanisms, but improvements to these strategies is needed
- Research is required to optimize post-exercise recovery with a focus on the brain

Post-exercise recovery has largely focused on peripheral mechanisms of fatigue, but there is growing acceptance that fatigue is also contributed to through central mechanisms which demands that attention should be paid to optimizing recovery of the brain. In this narrative review we assemble evidence for the role that many currently utilized recovery strategies may have on the brain, as well as potential mechanisms for their action. The review provides discussion of how common nutritional strategies as well as physical modalities and methods to reduce mental fatigue are likely to interact with the brain, and offer an opportunity for subsequent improved performance. We aim to highlight the fact that many recovery strategies have been designed with the periphery in mind, and that refinement of current methods are likely to provide improvements in minimizing brain fatigue. Whilst we offer a number of recommendations, it is evident that there are many opportunities for improving the research, and practical guidelines in this area.

## Introduction

Recovery from exercise is the process whereby the body is returned to a pre-exercise state (Halson and Jeukendrup, 2004; Barnett, 2006). The recovery process is of particular importance to athletes who are required to perform optimally over subsequent training sessions and competitions (Barnett, 2006). Although recovery from exercise occurs naturally over time, there is a desire by athletes and coaches to accelerate this process where time between exercise bouts is limited. As such, incorporating strategies to enhance recovery are now accepted as an important component of an athlete's training and competition program.

Outside of the sports psychology realm, post-exercise recovery literature has almost exclusively focused on the recuperation of peripheral fatigue. However, performance is also reliant on central processes, which have been implicated in fatigue since the late 19th century (Waller, 1891; Mosso, 1914). Practitioners, coaches, and athletes now accept that recovery is also for the mind, summed up by successful British Athletics coach Frank Dick:

*When you've driven yourself to exhaustion, it is seldom only physical. It's also mental and emotional. So real recovery must address all three* - @frankdickcoach, 21st November 2013.

Despite this anecdotal acceptance, little research has investigated how contemporary recovery strategies impact on the brain. Although it appears that restoration of peripheral fatigue will improve afferent feedback, recovery techniques have also been shown to directly affect the brain. Recently, Minett and Duffield (2014) highlighted the need to ameliorate central nervous system (CNS; including the brain) related fatigue during recovery but provided little insight into how this might occur. In this narrative review, the potential for common recovery practices to assist in brain recovery are highlighted. Although a largely theoretical review, in many cases there is not sufficient depth in the literature, if any, addressing fatigue and recovery on the brain. It may be argued that feed forward and feedback mechanisms play a role in fatigue and subsequent recovery, but this is not likely to explain all of the mechanisms at play. In fact research claiming the success of peripheral recovery strategies cannot dismiss the possibility of the brain contributing to or explain the results. The aim of this review however is not to argue against peripheral fatigue, but rather raise awareness of fatigue in the brain, and the implications for its subsequent recovery. To do so we highlight many of the common recovery strategies adopted by athletes, and point out how these may have direct effects on the brain. The research reviewed focuses on modalities that improve performance, since little research exists examining the brain directly. We make the case that future research should focus on better understanding the role of the brain in recovery, and offer some thoughts on future research in this area.

## Definitions and background

Fatigue is a complex concept often debated and has been defined simply as an inability to maintain a power output or force during repeated muscle contractions (Gibson and Edwards, 1985). Whilst this definition has academic merits to assist in explaining descending motor drive, in terms of exercise or sporting performance, it lacks the sensitivity of detecting other manifestations of fatigue in the brain, such as altered decision-making, mood disturbances, decreases in responding to opposition cues, reductions in skill execution or motivation changes. These and other factors are also important for the athlete and form a type of fatigue based predominantly in the brain that athletic training aims to build a resilience toward, and recovery practices should aim to ameliorate prior to subsequent performance. Previous works offer comprehensive insight into the role of the CNS in muscle fatigue (e.g., Gandevia, 2001), but our focus remains on the brain, largely upstream of the motor cortex. In this regard, the psychobiological model (Smirmaul et al., 2013) presents as a better model through which brain fatigue in an exercise context could be assessed. Psychobiology however could refer to any interaction between psychology and the biological state of (a part of) the brain and it is under this slightly broader definition that we argue for improving recovery strategies as it relates to exercise and sporting performance.

## Nutritional aids

### Carbohydrates

Carbohydrate feeding is perhaps one of the most well recognized recovery strategies post-exercise. Research on the muscle biopsies of endurance athletes has provided strong evidence of carbohydrate utilization during exercise, and that without adequate carbohydrate restoration, subsequent performance is impaired, associated with reduced muscle glycogen stores (Karlsson and Saltin, 1971). The recommendations for carbohydrate intake during and post-exercise are now well established and largely based on observations of glucose uptake rates, as well as skeletal muscle biopsies (Jentjens and Jeukendrup, 2003; Burke et al., 2004). There is little doubt however that carbohydrate will affect performance by way of the brain through diverse mechanisms. For instance, recent research has revealed that carbohydrate in the mouth has an immediate effect on fatigue. Mouth sensing of carbohydrate immediately improves motor output and force generation, minutes prior to elevating blood glucose (Gant et al., 2010), but these improvements dissipate in individuals who are already fed (Rollo and Williams, 2010). Carbohydrate is clearly important to the brain if anticipatory mechanisms impact performance, but it is the more direct influence that is better understood.

Carbohydrate may act through influencing neurotransmitter precursors that cross the blood-brain barrier. The neurotransmitter serotonin for instance is influenced by the levels of its precursor, tryptophan. Tryptophan is transported across the blood-brain barrier when in its free state, but can be taken out of free circulation by binding to albumin. Albumin can be theoretically manipulated by carbohydrate ingestion through its effect on lipolysis, and concomitant reduction in circulating fatty acid concentration which has a higher affinity for albumin than does tryptophan. Indeed carbohydrate ingestion reduces the amount of tryptophan crossing the blood-brain barrier during prolonged exercise (Blomstrand et al., 2015) in a dose dependent manner (6 or 12% carbohydrate solution) (Davis et al., 1992). More recently however, animal studies suggest that extracellular glucose may restrict the rise in exercise induced serotonin, not through synthesis, but rather through regulating its release and reuptake (Bequet et al., 2002). Regardless, serotonin is of interest as it has been associated with depression, sleepiness, and mood (Strüder and Weicker, 2001a,b) although the ratio of serotonin to dopamine, as well as other neurotransmitters, may relate more closely with central fatigue (Meeusen et al., 2006; Meeusen and Watson, 2007). Carbohydrate uptake post-exercise may therefore improve fatigue and mood although, to the authors' knowledge, there is no research investigating how carbohydrate specifically influences serotonin level into the post-exercise recovery period.

Neurotransmission may also be influenced by by-products of metabolism in muscle. The metabolic by-product ammonia can have an effect on brain fatigue through its interference with cerebral energy metabolism and neurotransmission (Wilkinson et al., 2010). Circulating ammonia increases with exercise intensity and duration as fuel stores deplete. Addressing this potential muscle fuel crisis, carbohydrate ingestion during prolonged exercise reduces levels of ammonia in cerebrospinal fluid (Nybo et al., 2015). Although current direct evidence between exercise-elevated ammonia and fatigue is weak, carbohydrates influence on ammonia may be another avenue with which post-exercise recovery can be promoted.

The beneficial effects of carbohydrate intake post-exercise are also likely to relate to the replenishment of brain glycogen used as a fuel source during exercise. Brain glycogen is confined to the astrocytes which serve to nourish surrounding neurons. Astrocyte glycogen levels reach 5–6 mM (Dalsgaard et al., 2006) in gray and white matter and higher in the hippocampus. Secher et al. (2008) suggest that given the volume of astrocytes in the brain, glycogen levels are similar to that observed in skeletal muscle. Research measuring lactate and glucose uptake across the brain show that there is a large carbohydrate uptake by the brain following demanding exercise, suggesting the importance of brain glycogen during at least some forms of exercise (Ide et al., 2000; Dalsgaard et al., 2002; Nybo et al., 2003). Animal studies confirm that brain glycogen is reduced following forms of exercise in which blood glucose (or lactate) is unable to keep up with the energetic demands of brain tissue (Choi et al., 2003). Rats undergoing 2 h of treadmill running at moderate intensity results in ~37–60% reduction in brain glycogen, localized in regions of the brain likely to be more engaged in exercise regulation (Matsui et al., 2011).

It has been established that muscle increases its glycogen content in response to a training stimulus, and it appears that this also occurs within the brain (Choi et al., 2003; Matsui et al., 2012). Utilizing an injectable glucose solution post-exercise, in which brain glycogen levels decreased by 50–64%, Matsui et al. (2012) showed that brain glycogen supercompensated by 29–63% (whole brain 46%, cortex 60%, hippocampus 33%, hypothalamus 29%, cerebellum 63%, and brainstem 49%) at 6 h after exercise. In cortical and hippocampal tissue, glycogen supercompensation was sustained until 24 h post-exercise. It has been suggested previously that it may be possible to increase brain glycogen stores and Gailliot (2008) points to examples of both direct and indirect evidence that this is the case. More recently this was confirmed with the observation that basal glycogen levels in the cortex and hippocampus increase with 4 weeks of exercise training (Matsui et al., 2012). These adaptations are similar to the pattern of response described after exercise for muscle glycogen, suggesting that the brain may benefit from similar interventional strategies.

It is also likely that carbohydrate feeding post-exercise will improve the athletes' psychological state as supplemental carbohydrate over a physically demanding day improves vigilance and mood (Lieberman et al., 2002). This may be due to the link between carbohydrate intake and serotonin or a direct consequence of brain glycogen levels, or both. In a similar fashion, carbohydrate intake is likely to assist in executive functioning (Gailliot, 2008) post-exercise and it would seem probable that any intervention that improves life outside of sport would also be beneficial for an athlete's recovery.

There are clear avenues by which carbohydrate could assist the brain in the recovery process. There is however little research investigating these interactions and no doubt more potential mechanisms to uncover. For instance, the effects of carbohydrate replenishment on other important factors associated with brain health and performance, such as brain derived neutrophic factor, post-exercise remain unknown. In investigating carbohydrate and the brain however, it is likely to be problematic distinguishing the effects of carbohydrate feeding on the brain and the well-established peripheral effects. No doubt though, improved recovery strategies could be developed once we better understand these carbohydrate—brain performance pathways. A major limitation in this area is the technological limitations surrounding the measurement of glycogen in the brain. Although irradiation techniques are offering opportunities to investigate cerebral glycogen in animal models, the capacity to measure this in intact human models is only beginning to emerge. The development of ^13^C Magnetic Resonance Spectroscopy has recently allowed for the measurement of brain glycogen and turnover under physiological conditions (Khowaja et al., 2014) and further research with these tools in exercise settings are likely to vastly improve our understanding of the exercise fatigue and recovery within the brain.

### Protein

Branched chain amino acid (BCAA) supplementation is widely used among athletes in order to improve recovery and adaptation by maximizing net protein accretion following exercise, especially following resistance-type exercise (Tipton et al., 2001). However, it has been suggested that in addition to these peripheral factors, ingestion of BCAA may also minimize fatigue and improve recovery via central mechanisms (Blomstrand, 2006; Meeusen et al., 2006).

As briefly suggested above, serotonin is involved in feelings of lethargy, drowsiness, and loss of motivation and may play a role in fatigue during or following exercise (Blomstrand, 2006; Meeusen and Watson, 2007). The rate of serotonin synthesis in the brain is largely dependent on circulating levels of tryptophan which is transported across the blood-brain barrier as previously described (Meeusen and Watson, 2007). Because tryptophan is also mediated by the same carrier system as BCAA, manipulation of the ratio of free tryptophan to BCAA (e.g., via BCAA supplementation) can affect tryptophan movement across the blood-brain barrier, subsequently effecting serotonin production (Blomstrand, 2006; Meeusen and Watson, 2007).

The tryptophan-serotonin theory has been confirmed in animal models. Indeed, when a saline infusion (placebo) was delivered using an *in vivo* brain microdialysis technique, exercised rodents had a progressive increase in extracellular serotonin; whereas this increase was eliminated when rodents were given the BCAA L-valine prior to exercise (Gomez-Merino et al., 2001). Exercise capacity and voluntary running performance have also been improved following BCAA supplementation in animals (Calders et al., 1997, 1999; Smriga et al., 2002). Calders et al. (1999), reported rats injected intraperitoneally with BCAA 5 min prior to a run to exhaustion, ran significantly longer (158 ± 28 min) when compared to a saline injection (118 ± 35 min). Results in humans are more mixed and, although there are examples of improved performance with BCAA supplementation (Blomstrand et al., 1997; Mittleman et al., 1998), the majority of studies have reported no benefit of BCAA supplementation on performance (Varnier et al., 1994; Van Hall et al., 1995; Madsen et al., 1996; Strüder et al., 1998; Davis et al., 1999; Cheuvront et al., 2004).

One potential reason that BCAA displays limited, or no performance benefit may be that many BCAA preparations also reduce tyrosine uptake, and subsequently the synthesis and release of catecholamines, notably dopamine (Fernstrom, 2013). Dopamine stimulating drugs are known to enhance aspects of exercise performance (Roelands et al., 2008), thus BCAA administration may benefit from the addition of tyrosine in any supplementation routine. Certainly, acutely depleting plasma tyrosine results in reduced exercise capacity (Tumilty et al., 2013). Further work in this area is required.

Although the theory for supplementation of BCAA to minimize fatigue is appealing, the limited evidence is not convincing to suggest BCAA supplementation alone as a method to minimize brain fatigue. However, from an applied perspective, the improvements observed in ratings of perceived exertion (although not accompanied by improvements in performance) may be beneficial in some sporting environments and could be incorporated with other methods outlined in this review. Overcoming the depression of tyrosine with BCAA supplementation, affecting the dopamine system, may be a particular area of interest in the near future.

### Hydration

Hydration provides another area in which a homeostatic balance must be maintained, or returned to during recovery, and its effects on the periphery are well reported in the literature. Surprisingly however, this remains an emerging area of research for the brain (Adan, 2012) without consistent findings. It is believed that 2% dehydration impairs attention, psychomotor, and immediate memory skills, as well as assessment of the subjective state, providing a clear incentive for individuals to maintain, or return to a euhydrated state. Structurally, acute dehydration is thought to result in an overall shrinking of the brain (Kempton et al., 2009; Streitbürger et al., 2012), although not always (Watson et al., 2010). The direct effect of this on performance however is unknown, but a likely preservation or return to a euhydrated state during recovery is desirable. Cerebral blood flow is also compromised with dehydration, although this does not appear to affect the cerebral metabolic rate of oxygen (Trangmar et al., 2014), at least not whilst compensatory mechanisms can cope. Similarly, changes in the permeability of the blood-brain barrier have also been related to changes in hydration state (Watson et al., 2006) and may contribute to the development of central fatigue (Nierwińska et al., 2008). Changes to the blood-brain barrier during exercise appear fairly robust however, as attempts to observe changes in its permeability with endurance exercise in the heat have provided mixed results (Watson et al., 2005; Morrison et al., 2013). Thus it is not clear if the blood-brain barrier is susceptible to exercise-related exposure to heat, dehydration or a combination of both, although some evidence suggests that brain temperature is the over-riding factor (Kiyatkin and Sharma, 2009).

Hydration state may however alter the effort associated with subsequent efforts. In an MRI study, Kempton et al. (2011) found that, a period of dehydrating exercise resulted in greater neuronal activity in regions of the brain involved in cognitive tasks compared to a control exercise condition. Whilst the reduction of some of the cerebral parameters may not directly affect performance due to the compensation mechanisms protecting the brain, these coping mechanisms will not have endless capacity for compensation, and importantly, may not allow for the complete recovery of the system. As a case in point, brain glycogen is not thought to regenerate normally except during sleep, even though reductions in glycogen levels do not necessarily reduce cognitive (or physical) performance (see Sleep section). The return to a euhydrated state as quickly as possible in the post-exercise recovery period is therefore a clear goal for athletes, regardless of whether the effect is due to hydration *per se*, or through thermoregulatory means.

## Temperature regulation

The capacity to perform prolonged exercise is greatly reduced in a warm environment (Hargreaves and Febbraio, 1998). Exercise capacity is greatest in non-heat acclimated males at an ambient temperature of 11°C (Galloway and Maughan, 1997) and progressively declines as ambient temperature increases (Parkin et al., 1999). As such, recovery interventions which affect the regulation of body temperature are commonly used during and after exercise in the heat (Casa et al., 2007; Vaile et al., 2008a; Stanley et al., 2010; Hausswirth et al., 2012; Minett et al., 2012, 2013; Pointon et al., 2012). Immersion in cold water (CWI) is regularly used post-exercise to treat the symptoms of exercise induced muscle damage but is also regarded as the most effective method of treating exercise induced heat stress (Casa et al., 2007). More recently methods such as ice slushie ingestion (Stanley et al., 2010), cooling vests (Hausswirth et al., 2012; Minett et al., 2012) and cold towels (Minett et al., 2012) have also been studied as a convenient way to treat exercise induced thermoregulatory strain, although with mixed results. Whilst the effects of hyperthermia on exercise in the heat have been shown to be reasonably clear, their mechanisms are not entirely understood. Often, reduced exercise capacity is attributed to the attainment of a critical core temperature. In elite cyclists performing a 30 min self-paced time trial, mean power was reduced by 6.5% in the heat. Interestingly though, rectal temperature (a measure of core temperature) was similar between trials, and blood lactate was lower at the end of the warm trial. As impaired substrate availability, dehydration or lactate accumulation does not explain the declines in performance, the role of the brain in regulating exercise during the heat has been proposed as an important factor (Nybo, 2010). Exercise in the heat has been shown to have a direct impact on brain activity. Hyperthermia causes a progressive reduction in pre-frontal cortex activity, as evidenced by an increase in the ratio of α–β frequency bands from an electroencephalogram recording (Nielsen et al., 2001). The shift toward lower frequency α bands is characterized by feelings of drowsiness and a loss of motivation normally experienced when transitioning from a fully alert state to sleep (Nielsen et al., 2001; Meeusen et al., 2006). Therefore, it would seem that recovery from thermoregulatory strain is important, from a brain perspective as well as in the periphery, prior to subsequent exercise performance.

Brain temperature is thought to remain ~0.2°C above core temperature (Nybo et al., 2002b), thus any challenge to thermoregulation also affects the brain. The brains thermoregulatory center, the hypothalamus, controls the thermoregulatory reflexes via afferent and efferent signals which may explain the shift toward low frequency α bands during exercise in the heat. As serotonergic and catecholaminergic projections innervate the hypothalamus, changes in the activity of these neurons would also be expected to contribute to the control of body temperature throughout both exercise and rest. Similarly, heat stress during exercise reduces cerebral blood flow and oxygenation which is suggested to alter central motor output (Nybo et al., 2002a). Attempts to selectively cool the brain remain relatively unsuccessful (e.g., Nybo et al., 2014), and it appears as though global lowering of body temperature is required, as arterial blood will gradually lower brain temperature (Nybo et al., 2002b). CWI increases mean arterial pressure and cardiac output, which should result in an increase in cerebral oxygenation (Vaile et al., 2008a). However, Minett et al. (2013) found CWI decreased cerebral oxygenation, as measured by near-infrared spectroscopy, after exercise in the heat despite improved recovery of neuromuscular function and exercise performance. Oxygenation in the pre-frontal cortex, as measured by Minett et al. (2013), may not however detect changes that occur with CWI in other brain regions. After prolonged exercise in the heat, in which β activity decreased across the whole brain, De Pauw et al. (2013) subjected participants to different recovery strategies including CWI. The authors observed an increase in β (β_3_) activity only after CWI, and not after recovery strategies that did not target thermoregulatory mechanisms. Increases in β activity however were restricted to brain areas involved in somatosensory information processing. Cold water will obviously induce a somatosensory response, but warm water may also influence cerebral activity. Although not providing a strong thermoregulatory response, the act of immersing in warm water has been shown to increase cerebral blood flow velocity (Carter et al., 2014) and may therefore provide a mechanism by which recovery is aided post competition. Thus recovery methods such as CWI (and water immersion in general) may offer a number of mechanisms by which the brain regulates exercise performance such as changes in regional blood flow, alterations in psychological state and changes in inhibitory and stimulatory pathways that precede the descending motor drive.

The effects of increased thermoregulatory strain on exercise performance are very clear and more is now understood on the contribution of the brain to fatigue. Recovering the brain after exercise in the heat is an area which requires further research for not only a sporting context but also the potential application to emergency services or armed forces. Due to this a better understanding of the effects of CWI on the brain as well as investigating methods which are easily and quickly applied, such as ice vests and slushies, is warranted.

## Inflammatory regulation

Inflammation is associated with muscle damage and plays a key role in the cascade of events that occur in order to repair a damaged muscle (Kharraz et al., 2013). Because of this, levels of circulating pro- and anti-inflammatory cytokines are often used as measures of fatigue and recovery (Vaile et al., 2008b). Recovery strategies, such as CWI, cryotherapy, and nutritional strategies which have been shown to minimize post-exercise inflammation are therefore regularly incorporated into an athlete's training program in order to promote recovery.

As well as playing a role in muscle repair, inflammatory cytokines signal the brain through several immune-to-brain communication pathways which result in central neural changes and associated behavioral alterations such as feelings of tiredness and reduced motivation (Dantzer et al., 2014). In addition to these communication pathways, D'mello et al. (2009) identified that peripheral increases in immune cell production lead to active recruitment of monocytes in the brain. Increases in cerebral monocytes in mice resulted in significant reductions in social interaction time and increased inactivity compared with mice with cerebral monocytes inhibition (i.e., no increase in brain monocytes, but increased peripheral inflammation). These findings may suggest that the behavioral alterations observed are in part caused by both inflammatory related changes within the brain and communication to the brain.

Similar reductions in motivation have been observed when strong immune responses are elicited through mice treated with lipopolysaccharides. Lipopolysaccharides (large molecules used to elicit a strong immune response) can increase brain concentrations of IL-1-beta, part of the wider exercise-induced response particularly prevalent after unaccustomed, or eccentric-based exercise. In support of this, Carmichael et al. (2006) found that a bout of downhill running reduced subsequent running performance in mice, but this was overcome when an intracerebroventricular injection of the IL-1 receptor agonist was used 2 h prior to the run time to fatigue. Further, intracerebroventricular injection of IL-1-beta in a matched uphill running group reduced subsequent run time to fatigue to a similar extent, highlighting that IL-1 in the brain is the likely cause of early fatigue in these groups. Similar results have been observed in humans as 10 km running time trial performance is impaired in trained runners after the administration of recombinant IL-6 compared to placebo (Robson-Ansley et al., 2004). As such, performance improvements observed following recovery strategies which promote a reduction of inflammation, which is typically associated with peripheral fatigue, may also be due to improved motivation and subjective ratings. Indeed, Cook and Beaven (2013) reported that there is important role of individual perception in enhancing training recovery. Nutritional interventions such as BCAA (Matsumoto et al., 2009) and carbohydrate ingestion (Nehlsen-Cannarella et al., 1997; Nieman et al., 1998) have also been linked to reduced inflammation post-exercise representing yet more cross-over between recovery strategies although links to brain fatigue have not been conducted in this context. It is unclear if inflammation has other manifestations on brain fatigue related indices and it may be that inflammation has other consequences on sporting performance.

The mitigation of inflammation has implications for sport science research, especially where maximal effort is expected but little incentive is given. Based on the suggestions above, some of the performance benefits from recovery strategies that minimize inflammation may only be evident in research-based environments, or training scenarios, as, during actual competitive performance the incentive may be great enough to counteract the reduced motivation performance decrements. Regardless, reductions in motivation can be seen as a form of brain fatigue and if exercise or sporting performance is to remain optimal, recovery strategies should also aim to reduce inflammation.

## Sleep

It has long been established that sleep deprivation may lead to impaired psychological, physiological and cognitive function when it comes to sporting performance (Thomas et al., 2000). Restricted or disturbed sleep in elite athletes is associated with impairments in mood and motivation (Sinnerton and Reilly, 1992), compromised immune function (Pyne et al., 2000) and symptoms of overreaching (Fry et al., 1994; Jeukendrup and Hesselink, 1994). In several recent studies evaluating the sleeping habits of elite athletes, it was reported that on average, athletes get ~6.5–7.2 h sleep each night, significantly less than their non-athletic counterparts (Teng et al., 2011; Leeder et al., 2012; Juliff et al., 2015). Conversely, increasing the amount of sleep an elite athlete obtains to 8 h or more per night can significantly improve sports-specific performance (Mah et al., 2011). Sleep is often referred to as being one of the key strategies for athletes to achieve both mental and physical recovery following exercise (Halson, 2008) suggesting that quality sleep is a critical component for sporting success.

While there are very few studies to examine sleep as a recovery strategy following exercise, there has been a plethora of research performed to assess changes in brain function associated with sleep deprivation (Durmer and Dinges, 2005). The first documented experimental study of the cognitive performance effects of sleep deprivation on humans was reported in 1896, involving three participants subjected to 90 h of continuous wakefulness (Patrick and Gilbert, 1896). Virtually all forms of sleep deprivation result in increased negative mood states, especially feelings of fatigue, loss of vigor, sleepiness, and confusion (Durmer and Dinges, 2005). Moreover, it was revealed in a meta-analysis of 143 sleep deprivation studies, that mood is more affected by sleep deprivation than both cognitive and motor performance (Pilcher and Huffcutt, 1996). In brain imaging studies using positron emission tomography (PET) scans, depressed mood states are easily identified in the prefrontal cortex, and more specifically in the lateral, orbitofrontal, and ventromedial regions (Baker et al., 1997). These negative mood states often present as an overall reduction in brain activity in these regions. The prefrontal cortex is known not only to be involved in mood responses, but also to have numerous connections with other parts of the brain that are responsible for controlling dopamine, norepinephrine and serotonin, three neurotransmitters that are important in mood regulation (Ruhé et al., 2007). Therefore, a clear link can be made between sleep deprivation and the neurochemistry of negative mood states in the brain. Of course, the inverse relationship can be assumed. If athletes are to obtain better quality and quantity of sleep, we can expect this to contribute to a better mood, and an overall enhanced recovery process.

Theories of how sleep deprivation affects cognitive abilities are ever evolving as both the range of cognitive effects from sleep loss and the neurobiology of sleep-wake patterns are better understood. Recent experiments reveal that following days of chronic sleep restriction, significant daytime cognitive dysfunction accumulates to levels comparable to that found after severe acute total sleep deprivation (Durmer and Dinges, 2005). In a military study where subjects were deprived of sleep for over 72 h, How et al. (1994) employed a battery of tests related to cognitive and physical performance. The more pronounced declines were observed in cognition, speed and precision while smaller effects were found in routine tests of physical measures. Furthermore, a meta-analysis of relevant studies has confirmed the significant impact of sleep deprivation on psychomotor/cognitive performance. Koslowsky and Babkoff (1992) concluded that the longer the period without sleep, the greater was the effect on cognitive ability. The researchers also highlighted that decreases in speed were greater than decrements in accuracy. It is likely that decreases in psychomotor/cognitive performance with sleep loss are related to the function of brain signaling and metabolism and further highlights the importance of sleep in the recovery of athletes.

Brain function in the prefrontal cortex, the anterior cingulate and the posterior parietal systems seem particularly vulnerable to sleep loss (Chuah et al., 2006). Regional brain activation studies using PET (Wu et al., 1991; Thomas et al., 2000) and functional magnetic resonance imaging (fMRI) (Portas et al., 1998; Drummond et al., 1999, 2001) show changes in response to sleep deprivation. PET studies show a global decrease in glucose metabolism throughout cortical and subcortical regions during sleep deprivation. As individuals become impaired on cognitive tasks, a more specific decrease in glucose uptake occurs in the prefrontal cortex, thalamus, and posterior parietal association cortices (Thomas et al., 2000). fMRI studies indicate that after 24 h of total sleep deprivation, attention-demanding tasks demonstrate increases in thalamic activation (Portas et al., 1998). It is these impairments in the metabolic functions of the brain that may contribute to the cognitive and/or psychomotor dysfunction when sleep loss is apparent. Decreases in glucose metabolism are a likely consequence of reductions in brain glycogen (discussed earlier) which are observed when sleep is compromised (Kong et al., 2002; Brown, 2004). The ability of sleep to restore brain glycogen has been readily accepted for decades (Brown, 2004) but it is interesting from an applied perspective that brain glycogen may accumulate very slowly with sleep, perhaps taking up to 9 h to restore to baseline levels after a period of sleep deprivation. Brain glycogen may not be the only concern following sleep deprivation as recently it has been observed that sleep plays an important role in cleansing the brain of neurotoxic waste (Xie et al., 2013) as well as in gene regulation (Archer et al., 2014). The consequences of altering these sleep dependent actions on physical and mental performance are however unknown.

Recommendations for good sleep hygiene practices in the literature typically include the creation of a cool and dark sleep environment, avoiding electronics in the bedroom, abstaining from caffeine in the latter half of the day and maintaining a routine of going to bed, and waking, at a similar time each day. There is also potential for interactions between sleep quality and other recovery interventions. For instance, sleep onset coincides with a drop in body temperature (Murphy and Campbell, 1997) following the normal circadian rhythm. Interventions manipulating body temperature may therefore also assist with sleep although recent post-exercise CWI studies have had no effect on sleep quality, quantity (Robey et al., 2013a) or melatonin (Robey et al., 2013a,b). Subtle manipulations that increase skin temperature however have been shown to improve sleep (Raymann et al., 2008). A limited amount of research has also investigated the effects of different foods on sleep quality but indications are that carbohydrate and protein intake, as well as some micronutrients (e.g., nitrates, tryptophan and melatonin), influence different sleep characteristics such as sleep latency and sleep quality (see Halson, 2013). Further detail on sleep and nutritional interactions in elite athletes are available in a recent review (Halson, 2014). As mentioned previously, technological limitations have prevented the assessment of how carbohydrate intake timing, type and quantity prior to sleep influences the restoration of brain glycogen, and indeed, to what extent sleep modulates this restoration.

While the effect of sleep on recovery in the brain following exercise remains largely unknown, we have a comprehensive understanding of what sleep loss/deprivation does to overall brain function. The impairment of brain function with sleep deprivation expresses itself through poor performance in cognitive/psychomotor skills, mood, and motivation. All of these factors are vital to sports performance at any level of competition. Therefore, it can be assumed that an enhanced quality and/or quantity of sleep may aid in performance improvements. Given the reports of poor sleep quality in elite athletes, more research is required to examine the relationship between sleep as a recovery strategy post-exercise and both physical and mental performance.

## Recovery from mental fatigue

One area almost entirely overlooked in the recovery of an athlete is the importance of recovery from mental fatigue. Mental fatigue is a change in psychophysiological state, caused by prolonged periods of demanding cognitive activity (Marcora et al., 2009) and this change is gradual and cumulative and can include increased resistance against further effort (Meijman, 2000), changes in mood (Broadbent, 1979; Holding, 1983) and feelings of “tiredness” and “lack of energy” (Boksem and Tops, 2008). Mental fatigue can be brought about by the sustained performance of a single cognitive task but importantly can also include different tasks that require mental effort, such as fatigue incurred through a working day. The effect of mental fatigue on cognitive performance is well known, however more recently mental fatigue has reduced time to exhaustion during high-intensity cycling (Marcora et al., 2009), reduced average running speed during a 5 km running time trial (Pageaux et al., 2014) and increased the perception of effort during a prolonged submaximal isometric contraction (Pageaux et al., 2013). Despite the relatively consistent observation that mental fatigue impairs subsequent endurance performance, the mechanisms behind this effect is presently unknown. Previous studies have revealed no difference in any physiological variable between mental fatigue and control conditions, the single discrepancy between trials the greater perceived exertion (RPE) experienced by mentally fatigued participants (Michalsen et al., 2005; Marcora et al., 2009; Pageaux et al., 2013). Prolonged mental exertion is hypothesized to directly affect the cortical centers involved in the cognitive aspect of central motor command (Hallett, 2007), and the primary sensory input for perceived exertion (Marcora, 2009). Specifically the anterior cingulate cortex (ACC), an area of the prefrontal cortex strongly activated by cognitive effort (Paus et al., 1998). These findings support the involvement of the ACC in mental fatigue as correlations have been observed between changes in ACC activation and changes in RPE during manipulations of exercise intensity under hypnosis and motor imagery (Williamson et al., 2001, 2002, 2006), and rats with experimental ACC lesions engage significantly less than normal rats in tasks requiring physical effort to obtain a larger reward (Walton et al., 2003, 2006; Rudebeck et al., 2006). Furthermore, experimental evidence from *in vitro* and animal studies suggest that neural activity increases extracellular concentrations of adenosine (Karlsson and Saltin, 1971), and that brain adenosine induces a reduction in endurance performance (Burke et al., 2004). Cognitive task-induced adenosine accumulation in the ACC has also been hypothesized to mediate the increased perception of effort (Pageaux et al., 2014).

To date the reduced endurance performance in a mentally fatigued state, has been attempted to be explained by the psychobiological model of exercise tolerance (Marcora, 2008; Marcora et al., 2008). This model proposes that a time-to-exhaustion test is determined primarily by two cognitive factors: perceived exertion and potential motivation. In other words, participants decide to “give up” either because the effort required to complete the task exceeded the greatest effort they were willing to exert in order to succeed, or because the effort to complete the task was so high that continuing for any longer is beyond their perceived ability (Wright, 1998). Consequently, exercise performance may improve or reduce, relative to the changes in perceived exertion or motivation. Aside from accounting for the reduced performance of mentally fatigued participants, this model rationalizes the reduced RPE and hence improved cycling time trial performance of athletes using a glucose mouthwash (Chambers et al., 2009) and the greater power output during a RPE matched cycling time trial following amphetamine ingestion (Swart, 2009). Similarly motivational self-talk (Blanchfield et al., 2013), and the presence of an attractive female “research assistant” (Winchester et al., 2012) reduced ratings of perceived exertion during matched workload exercise trials. Most astonishingly, the mere suggestion of either an “uphill or downhill” grade during cycling under hypnosis was enough to significantly lower RPE during the imagined “downhill” and similarly elevate RPE during “uphill” cycling (Williamson et al., 2001). It is therefore apparent that any physiological or psychological factor affecting perception of effort or motivation may play a role in overcoming the detrimental effects of mental fatigue.

The link between recovery strategies, which aim to restore performance to pre-fatigue levels, and ergogenic aids, seeking to improve existing performance, is often blurred and in fatigue associated with the brain, this may be particularly true for caffeine. In the case of mental fatigue, caffeine may ameliorate, at least transiently, some of the reductions in subsequent exercise performance. Caffeine acts as a stimulant, and has been shown to reduce feelings of pain (Gailliot, 2008) and perception of effort (French et al., 2008) during exercise trials. Perhaps more importantly, the effects of caffeine are credited primarily to the inhibition of adenosine, by binding to the adenosine receptors in the CNS (Lorist and Tops, 2003). Accumulation of adenosine in the ACC has been hypothesized to contribute to the greater ratings of perceived exertion, and subsequent reduced endurance performance. As no further physiological mechanisms have been offered for the negative impact of mental fatigue on exercise performance, supplementation of caffeine may mediate this effect. This reduction in adenosine also leads to increased activity of the neurotransmitter dopamine (Lorist and Tops, 2003). Decreased secretion of dopamine is thought to underlie fatigue and impaired attention in polio survivors (Bruno and Zimmerman, 2000) and based on animal studies has been proposed to be central in acute fatigue, by regulating the tendency for expending energy, based on a cost/benefit analysis (Neill and Justice, 1981; Szechtman et al., 1994; Salamone et al., 1999). Furthermore methylphenidate-induced increases in dopamine concentration have been associated with increased task interest and motivation (Gant et al., 2010) and has been used effectively in the treatment of apathy, which is an extreme state of lack of motivation (Gill et al., 2006). The explicit use of caffeine to recover exercise performance in a mentally fatigued state however, is yet to be experimentally tested.

Another concept in its early stages of research is the use of non-invasive brain stimulation techniques including transcranial direct current stimulation (tDCS) to alter perception of pain (Fregni et al., 2006), fatigue (Cogiamanian et al., 2007) and perceived exertion during exercise (Okano et al., 2013). Non-invasive brain stimulation techniques use an electrical current to stimulate specific parts of the brain, resulting in a polarity dependent modulation of brain activity. Okano et al. (2013) found RPE increased at a slower rate in 10 trained cyclists during an incremental test, following 20 mins of anodal tDCS applied over the left temporal cortex. Correspondingly, peak power output was also improved by ≈4%. However, not all research involving brain stimulation has produced positive results. Transcranial direct current stimulation applied to the motor cortex for 10 mins prior to an isometric elbow flexion task, did not alter maximum voluntary force or perception of effort (Lampropoulou and Nowicky, 2013). Hence further research in this area is warranted.

As well as the modalities of manipulation mentioned in this section, cognitive activity has a metabolic cost (Parks et al., 1988) and therefore the repletion of glycogen, along with the restorative properties of sleep mentioned in the sections above will play a role in the recovery of mental fatigue. Mental fatigue has an obvious detrimental effect on physical performance however no research has focused on recovery from such effects. Recovery strategies targeting changes in mood, feelings of fatigue, and perceived exertion would therefore no doubt assist in complete athlete recovery.

## Placebo effect

Recovery research often spruiks the role that the placebo effect may have on athletic performance but this was elegantly displayed recently by Broatch et al. (2014). The authors subjected participants to one of three recovery strategies immediately after a bout of high intensity efforts. Participants were led to believe that the addition of a substance to the thermo-neutral water in which they were immersed was beneficial for recovery, despite it being a non-descript skin cleanser. Strength performance was greater after the placebo recovery strategy compared to control, and similar to CWI. No physiological improvements were observed but psychological measures indicated that ratings of readiness for exercise, pain and vigor were all improved in the two conditions in which participants believed there would be a recovery benefit. Although this has historically been explained through theoretical models of suggestibility, physiological responses in the brain are now readily accepted as being able to explain, at least in part, the placebo effect. For instance, neuroimaging studies have revealed similar brain activation between placebo and pharmacological agents implicated with the opioid, cholecystokinin and dopamine neurotransmitter systems (Benedetti and Amanzio, 2013). A number of brain areas appear to respond to placebo including areas implicated with athletic performance including the prefrontal cortex (Watson et al., 2009; Lui et al., 2010) and ACC (Watson et al., 2009). Whilst many of these placebo observations relate to regions and systems associated with the perception of pain, offering the potential to regulate performance through pain modulation, the dopaminergic system offers a pathway in which reward experiences (and therefore motivation) could be manipulated (Benedetti and Amanzio, 2013).

The placebo effect then has a physiological role to play in the observed recovery of exercise performance. Placebo effects may explain the benefits observed from a large number of recovery strategies through similar pathways, but what is unclear, is how the performance improvement is dependent on the art of deception. Other researchers have shown that deception can work to an extent (Stone et al., 2012), although at some point there must be a limit to athletic performance. Psychological constructs such as deception, self-talk and similar are likely to influence physiological processes in the brain, but the extent, and durability of this approach will not be infinite, and we argue that the physiological recovery of brain homeostasis will be required for ongoing optimal performance. Further research is required to elucidate the placebo mechanisms further, as well as its limits, and how this can be taken advantage of in an athletic setting.

## Recommendations for brain recovery

**Figure 1:**
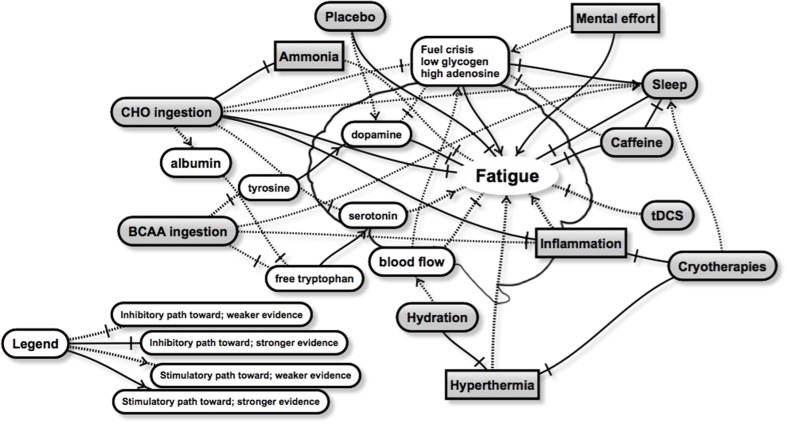
**A schematic representation of some of the interactions between a number of recovery strategies (rounded gray boxes) and factors related to brain fatigue**. Brain fatigue, manifesting through observations such as reduced muscle drive, changes in mood, reduced decision making or skill execution, or negative changes in motivation or perceived exertion may be overcome through various combinations of recovery strategies. The practical recommendations however remain unclear and will be the subject of future research. Further details in text.

Despite limited direct evidence of optimal recovery strategies for the brain, the current literature provides a number of possibilities for future research. We present a schematic highlighting some of the relationships between recovery strategies and brain fatigue (Figure 1). Whilst not exhaustive, the schematic aims to show the complexity of brain fatigue and the interrelationships involved, as well as areas of stronger and weaker evidence. From this schematic it appears that both carbohydrate and sleep present as the two major strategies to improve brain recovery, but a number of other strategies are also likely to contribute in a positive way. Regardless, the optimal timings, dose and combination with other recovery interventions remain unclear for brain recovery, and will attract future research. Whilst there will be inevitable improvements and discoveries relating to peripheral recovery, we believe that the greatest improvements in exercise recovery are likely to be found in strategies that directly target the brain, and optimize its recovery. Interventions that aim to restore fuel, such as nutritional strategies, as well as sleep are clearly likely to have the largest effect, although little is known on the effect size of any particular recovery strategy on the brain and its role in subsequent performance. Clearly, much more research is required in this area, and recommendations will be refined and changed as it becomes available. We hope that these insights will accelerate findings in this area.

## Recommendations for future research

There are perhaps two large impediments to developing knowledge in this area. The first is relating changes in performance to what occurs in the brain, as opposed to the periphery, and the second, is in assessing brain biology. As alluded to earlier, the role that the brain plays in fatigue, and therefore recovery, will be somewhat dependent on the model of fatigue being tested. Recent literature focuses heavily on the reduction of descending motor drive, or muscle power generated, in fatigue and recovery and this research provides valuable insights into elements of fatigue in the brain. However, more research is required adopting and testing fatigue and recovery using the psychobiological model of exercise performance. Further, the integration of psychological measures alongside physiological parameters as they relate to exercise performance are required, but no doubt much of this is due to the historic difficulties in evaluating brain biology in real time.

As suggested, measures likely to link to the psychobiological model should be more common place in exercise recovery research, and include appropriate and controlled measures of indices such as perceived exertion and motivation and its sub-scales. Measures such as concentration, attention, decision-making and others are also likely to provide further insight into brain fatigue and recovery within an exercise setting. Physiological measures within the brain are of course much more difficult to assess, but a number of technologies are available, some of which do not appear to have been adopted within the exercise literature, to assist in this area. The assessment of descending motor drive to drive muscle force generation is perhaps the best controlled methodology for assessing this manifestation of fatigue thanks largely to early seminal work articulated in earlier reviews (Gandevia, 2001). Newer technologies and others not used widely in the exercise literature provide potential to better understand fatigue in the brain. Improvements in the usability and development of mobile electroencephalogram (EEG) now allow for good quality measurement during physical activity (Reis et al., 2014). Whilst EEG is difficult to interpret, and cannot provide detail of neuronal activity in specific areas, changes in EEG activity are likely to continue to provide clues as to how brain physiology relates to psychology and exercise performance. Occupational and driver fatigue monitoring research utilizes methods such as EEG and pupil (diameter and response to bright lights) and ocular responses (including blink frequencies, velocity, and durations) and, although largely in the developmental stages (Dawson et al., 2014), provide promise for future research, especially as it relates to sleep related fatigue. Similarly, outside of more invasive animal research models, systemic humoral markers of energy balance, inflammatory responses and neutrophic factors will continue to provide clues as to how these relate to the brain in the absence of more direct measures *in-vivo*. Perhaps the most exciting opportunity at present however, one with a likely large effect on brain fatigue and recovery, is the development of 13C Magnetic Resonance Spectroscopy allowing for the measurement of brain glycogen and turnover under physiological conditions (Khowaja et al., 2014). These tools, combined with methodologies that assess different models of fatigue should greatly improve our understanding of fatigue in the brain, and its recovery.

Ultimately however it is the exercise and/or sporting performance that is the true test of an effective recovery strategy. Knicker et al. (2011) offer a number of alternative measures of fatigue that can exist in team sport environments, many of which could stem from inadequate recovery of the brain. Future research then will need to shift from the traditional assessments of exercise fatigue, and integrate new methodology in order to better assess the effectiveness of recovery strategies aimed at the exercise and sport performing brain.

## Conclusion

Existing evidence suggests that many commonly used post-exercise interventions aid in recovery of mechanisms of fatigue in the brain, and the peripheral manifestations normally targeted. Mechanistically, there is indirect evidence on how interventions aid brain recovery, though little research has investigated this area directly. We propose that the greatest advances in optimizing exercise recovery in the future will come from better understanding the brain. Future research should seek to better understand the effects of recovery modalities on the brain in order to optimize recovery post-exercise and ongoing improvements in imaging technologies are likely to contribute considerably to our understanding in this area. This research would contribute to improving global recovery recommendations, and to targeted strategies for individuals and their subsequent exercise adaption and performance above what is currently known for targeting peripheral recovery.

### Conflict of interest statement

The authors declare that the research was conducted in the absence of any commercial or financial relationships that could be construed as a potential conflict of interest.

## References

[B1] Adan A. (2012). Cognitive performance and dehydration. J. Am. Coll. Nutr. 31, 71–78. 10.1080/07315724.2012.10720011 22855911

[B2] Archer S. N. Laing E. E. Möller-Levet C. S. Van Der Veen D. R. Bucca G. Lazar A. S.. (2014). Mistimed sleep disrupts circadian regulation of the human transcriptome. Proc. Natl. Acad. Sci. U.S.A. 111, E682–E691. 10.1073/pnas.1316335111 24449876 PMC3926083

[B3] Baker S. C. Frith C. Dolan R. (1997). The interaction between mood and cognitive function studied with PET. Psychol. Med. 27, 565–578. 10.1017/S0033291797004856 9153677

[B4] Barnett A. (2006). Using recovery modalities between training sessions in elite athletes. Sports Medicine 36, 781–796. 10.2165/00007256-200636090-00005 16937953

[B5] Benedetti F. Amanzio M. (2013). Mechanisms of the placebo response. Pulmon. Pharmacol. Ther. 26, 520–523. 10.1016/j.pupt.2013.01.006 23370213

[B6] Bequet F. Gomez-Merino D. Berthelot M. Guezennec C. (2002). Evidence that brain glucose availability influences exercise−enhanced extracellular 5−HT level in hippocampus: a microdialysis study in exercising rats. Acta Physiol. Scand. 176, 65–69. 10.1046/j.1365-201X.2002.01015.x 12193220

[B7] Blanchfield A. W. Hardy J. De Morree H. M. Staiano W. Marcora S. M. (2013). Talking yourself out of exhaustion: the effects of self-talk on endurance performance. Med. Sci. Sports Exerc. 46, 998–1007. 10.1249/MSS.0000000000000184 24121242

[B8] Blomstrand E. (2006). A role for branched-chain amino acids in reducing central fatigue. J. Nutr. 136, 544S–547S. 16424144 10.1093/jn/136.2.544S

[B9] Blomstrand E. Hassmén P. Ek S. Ekblom B. Newsholme E. (1997). Influence of ingesting a solution of branched−chain amino acids on perceived exertion during exercise. Acta Physiol. Scand. 159, 41–49. 10.1046/j.1365-201X.1997.547327000.x 9124069

[B10] Blomstrand E. Møller K. Secher N. H. Nybo L. (2005). Effect of carbohydrate ingestion on brain exchange of amino acids during sustained exercise in human subjects. Acta Physiol. Scand. 185, 203–209. 10.1111/j.1365-201X.2005.01482.x 16218925

[B11] Boksem M. A. Tops M. (2008). Mental fatigue: costs and benefits. Brain Res. Rev. 59, 125–139. 10.1016/j.brainresrev.2008.07.001 18652844

[B12] Broadbent D. E. (1979). Is a fatigue test now possible? Ergonomics 22, 1277–1290. 10.1080/00140137908924702 540644

[B13] Broatch J. R. Petersen A. Bishop D. J. (2014). Postexercise cold-water immersion benefits are not greater than the placebo effect. Med. Sci. Sports Exerc. 46, 2139–2147. 10.1249/MSS.0000000000000348 24674975

[B14] Brown A. M. (2004). Brain glycogen re-awakened. J. Neurochem. 89, 537–552. 10.1111/j.1471-4159.2004.02421.x 15086511

[B15] Bruno R. L. Zimmerman J. R. (2000). Word finding difficulty as a post-polio sequelae. Am. J. Med. Rehabil. 79, 343–348. 10.1097/00002060-200007000-00005 10892620

[B16] Burke L. M. Kiens B. Ivy J. L. (2004). Carbohydrates and fat for training and recovery. J. Sports Sci. 22, 15–30. 10.1080/0264041031000140527 14971430

[B17] Calders P. Matthys D. Derave W. Pannier J.-L. (1999). Effect of branched-chain amino acids (BCAA), glucose, and glucose plus BCAA on endurance performance in rats. Med. Sci. Sports Exerc. 31, 583–587. 10.1097/00005768-199904000-00015 10211856

[B18] Calders P. Pannier J.-L. Matthys D. M. Lacroix E. M. (1997). Pre-exercise branched-chain amino acid administration increases endurance performance in rats. Med. Sci. Sports Exerc. 29, 1182–1186. 10.1097/00005768-199709000-00010 9309629

[B19] Carmichael M. D. Davis J. M. Murphy E. A. Brown A. S. Carson J. A. Mayer E. P.. (2006). Role of brain IL-1β on fatigue after exercise-induced muscle damage. Am. J. Physiol. Regul. Integr. Comp. Physiol. 291, R1344–R1348. 10.1152/ajpregu.00141.2006 16778069

[B20] Carter H. H. Spence A. L. Pugh C. J. Ainslie P. Naylor L. H. Green D. J. (2014). Cardiovascular responses to water immersion in humans: impact on cerebral perfusion. Am. J. Physiol. Regul. Integr. Comp. Physiol. 306, R636–R640. 10.1152/ajpregu.00516.2013 24553298 PMC4010659

[B21] Casa D. J. Mcdermott B. P. Lee E. C. Yeargin S. W. Armstrong L. E. Maresh C. M. (2007). Cold water immersion: the gold standard for exertional heatstroke treatment. Exerc. Sport Sci. Rev. 35, 141–149. 10.1097/jes.0b013e3180a02bec 17620933

[B22] Chambers E. S. Bridge M. W. Jones D. A. (2009). Carbohydrate sensing in the human mouth: effects on exercise performance and brain activity. J. Physiol. 587, 1779–1794. 10.1113/jphysiol.2008.164285 19237430 PMC2683964

[B23] Cheuvront S. N. Carter R. Kolka M. A. Lieberman H. R. Kellogg M. D. Sawka M. N. (2004). Branched-chain amino acid supplementation and human performance when hypohydrated in the heat. J. Appl. Physiol. 97, 1275–1282. 10.1152/japplphysiol.00357.2004 15358751

[B24] Choi I. Y. Seaquist E. R. Gruetter R. (2003). Effect of hypoglycemia on brain glycogen metabolism *in vivo*. J. Neurosci. Res. 72, 25–32. 10.1002/jnr.10574 12645076 PMC1471897

[B25] Chuah Y. L. Venkatraman V. Dinges D. F. Chee M. W. (2006). The neural basis of interindividual variability in inhibitory efficiency after sleep deprivation. J. Neurosci. 26, 7156–7162. 10.1523/JNEUROSCI.0906-06.2006 16822972 PMC6673955

[B26] Cogiamanian F. Marceglia S. Ardolino G. Barbieri S. Priori A. (2007). Improved isometric force endurance after transcranial direct current stimulation over the human motor cortical areas. Euro. J. Neurosci. 26, 242–249. 10.1111/j.1460-9568.2007.05633.x 17614951

[B27] Cook C. J. Beaven C. M. (2013). Individual perception of recovery is related to subsequent sprint performance. Br. J. Sports Med. 47, 1–5. 10.1136/bjsports-2012-091647 23293008

[B28] Dalsgaard M. K. Ide K. Cai Y. Quistorff B. Secher N. H. (2002). The intent to exercise influences the cerebral O_2_/carbohydrate uptake ratio in humans. J. Physiol. 540, 681–689. 10.1113/jphysiol.2001.013062 11956354 PMC2290259

[B29] Dalsgaard M. K. Madsen F. F. Secher N. H. Laursen H. Quistorff B. (2006). High glycogen levels in the hippocampus of patients with epilepsy. J. Cereb. Blood Flow Metab. 27, 1137–1141. 10.1038/sj.jcbfm.9600426 17133225

[B30] Dantzer R. Heijnen C. J. Kavelaars A. Laye S. Capuron L. (2014). The neuroimmune basis of fatigue. Trends Neurosci. 37, 39–46. 10.1016/j.tins.2013.10.003 24239063 PMC3889707

[B31] Davis J. M. Bailey S. P. Woods J. A. Galiano F. J. Hamilton M. T. Bartoli W. P. (1992). Effects of carbohydrate feedings on plasma free tryptophan and branched-chain amino acids during prolonged cycling. Eur. J. Appl. Physiol. Occup. Physiol. 65, 513–519. 10.1007/BF00602357 1483439

[B32] Davis J. Welsh R. De Volve K. Alderson N. (1999). Effects of branched-chain amino acids and carbohydrate on fatigue during intermittent, high-intensity running. Int. J. Sports Med. 20, 309–314. 10.1055/s-2007-971136 10452228

[B33] Dawson D. Searle A. K. Paterson J. L. (2014). Look before you (s) leep: evaluating the use of fatigue detection technologies within a fatigue risk management system for the road transport industry. Sleep Med. Rev. 18, 141–152. 10.1016/j.smrv.2013.03.003 23796506

[B34] De Pauw K. Roelands B. Maruši,è U. Tellez H. F. Knaepen K. Meeusen R. (2013). Brain mapping after prolonged cycling and during recovery in the heat. J. Appl. Physiol. 115, 1324–1331. 10.1152/japplphysiol.00633.2013 23990240 PMC3841834

[B35] D'mello C. Le T. Swain M. G. (2009). Cerebral microglia recruit monocytes into the brain in response to tumor necrosis factorα signaling during peripheral organ inflammation. J. Neurosci. 29, 2089–2102. 10.1523/JNEUROSCI.3567-08.2009 19228962 PMC6666330

[B36] Drummond S. Gillin J. C. Brown G. G. (2001). Increased cerebral response during a divided attention task following sleep deprivation. J. Sleep Res. 10, 85–92. 10.1046/j.1365-2869.2001.00245.x 11422722

[B37] Drummond S. P. Brown G. G. Stricker J. L. Buxton R. B. Wong E. C. Gillin J. C. (1999). Sleep deprivation-induced reduction in cortical functional response to serial subtraction. Neuroreport 10, 3745–3748. 10.1097/00001756-199912160-00004 10716202

[B38] Durmer J. S. Dinges D. F. (2005). Neurocognitive consequences of sleep deprivation. Semin. Neurol. 25, 117–129. 10.1055/s-2005-867080 15798944

[B39] Fernstrom J. D. (2013). Large neutral amino acids: dietary effects on brain neurochemistry and function. Amino Acids 45, 419–430. 10.1007/s00726-012-1330-y 22677921

[B40] Fregni F. Gimenes R. Valle A. C. Ferreira M. J. Rocha R. R. Natalle L.. (2006). A randomized, sham−controlled, proof of principle study of transcranial direct current stimulation for the treatment of pain in fibromyalgia. Arthritis Rheum. 54, 3988–3998. 10.1002/art.22195 17133529

[B41] French D. N. Thompson K. G. Garland S. W. Barnes C. A. Portas M. D. Hood P. E.. (2008). The effects of contrast bathing and compression therapy on muscular performance. Med. Sci. Sports Exerc. 40, 1297–1306. 10.1249/MSS.0b013e31816b10d5 18580411

[B42] Fry R. Grove J. Morton A. Zeroni P. Gaudieri S. Keast D. (1994). Psychological and immunological correlates of acute overtraining. Br. J. Sports Med. 28, 241–246. 10.1136/bjsm.28.4.241 7894955 PMC1332084

[B43] Gailliot M. T. (2008). Unlocking the energy dynamics of executive functioning: linking executive functioning to brain glycogen. Perspect. Psychol. Sci. 3, 245–263 10.1111/j.1745-6924.2008.00077.x 26158946

[B44] Galloway S. D. Maughan R. J. (1997). Effects of ambient temperature on the capacity to perform prolonged cycle exercise in man. Med. Sci. Sports Exerc. 29, 1240–1249. 10.1097/00005768-199709000-00018 9309637

[B45] Gandevia S. (2001). Spinal and supraspinal factors in human muscle fatigue. Physiol. Rev. 81, 1725–1789. 11581501 10.1152/physrev.2001.81.4.1725

[B46] Gant N. Stinear C. M. Byblow W. D. (2010). Carbohydrate in the mouth immediately facilitates motor output. Brain Res. 1350, 151–158. 10.1016/j.brainres.2010.04.004 20388497

[B47] Gibson H. Edwards R. (1985). Muscular exercise and fatigue. Sports Med. 2, 120–132. 10.2165/00007256-198502020-00004 3847097

[B48] Gill N. Beaven C. Cook C. (2006). Effectiveness of post-match recovery strategies in rugby players. Br. J. Sports Med. 40, 260–263. 10.1136/bjsm.2005.022483 16505085 PMC2491972

[B49] Gomez-Merino D. Bequet F. Berthelot M. Riverain S. Chennaoui M. Guezennec C. (2001). Evidence that the branched-chain amino acid L-valine prevents exercise-induced release of 5-HT in rat hippocampus. Int. J. Sports Med. 22, 317–322. 10.1055/s-2001-15645 11510866

[B50] Hallett M. (2007). Volitional control of movement: the physiology of free will. Clin. Neurophysiol. 118, 1179–1192. 10.1016/j.clinph.2007.03.019 17466580 PMC1950571

[B51] Halson S. L. (2008). Nutrition, sleep and recovery. Eur. J. Sport Sci. 8, 119–126 10.1080/17461390801954794

[B52] Halson S. L. (2013). Nutritional interventions to enhance sleep. Sports Sci. 26, 1–5. 10.1007/s40279-014-0147-0 PMC4008810 24791913

[B53] Halson S. L. (2014). Sleep in elite athletes and nutritional interventions to enhance sleep. Sports Med. 44, 13–23. 10.1007/s40279-014-0147-0 24791913 PMC4008810

[B54] Halson S. L. Jeukendrup A. E. (2004). Does overtraining exist? Sports Med. 34, 967–981. 10.2165/00007256-200434140-00003 15571428

[B55] Hargreaves M. Febbraio M. (1998). Limits to exercise performance in the heat. Int. J. Sports Med. 19, S115–116. 10.1055/s-2007-971973 9694414

[B56] Hausswirth C. Duffield R. Pournot H. Bieuzen F. Louis J. Brisswalter J.. (2012). Postexercise cooling interventions and the effects on exercise-induced heat stress in a temperate environment. Appl. Physiol. Nutr. Metab. 37, 965–975. 10.1139/h2012-077 22827512

[B57] Holding D. (1983). Fatigue, in Stress and Fatigue in Human Performance, ed Hockey R. (Durham: John Wiley and Sons).

[B58] How J. Foo S. Low E. Wong T. Vijayan A. Siew M.. (1994). Effects of sleep deprivation on performance of Naval seamen: I. Total sleep deprivation on performance. Ann. Acad. Med. Singap. 23, 669–675. 7847745

[B59] Ide K. Schmalbruch I. K. Quistorff B. Horn A. Secher N. H. (2000). Lactate, glucose and O_2_ uptake in human brain during recovery from maximal exercise. J. Physiol. 522, 159–164. 10.1111/j.1469-7793.2000.t01-2-00159.xm 10618160 PMC2269743

[B60] Jentjens R. Jeukendrup A. E. (2003). Determinants of post-exercise glycogen synthesis during short-term recovery. Sports Med. 33, 117–144. 10.2165/00007256-200333020-00004 12617691

[B61] Jeukendrup A. Hesselink M. (1994). Overtraining–what do lactate curves tell us? Br. J. Sports Med. 28, 239–240. 10.1136/bjsm.28.4.239 7894954 PMC1332083

[B62] Juliff L. E. Halson S. L. Peiffer J. J. (2015). Understanding sleep disturbance in athletes prior to important competitions. J. Sci. Med. Sport 18, 13–18. 10.1016/j.jsams.2014.02.007 24629327

[B63] Karlsson J. Saltin B. (1971). Diet, muscle glycogen, and endurance performance. J. Appl. Physiol. 31, 203–206. 5558241 10.1152/jappl.1971.31.2.203

[B64] Kempton M. J. Ettinger U. Foster R. Williams S. C. Calvert G. A. Hampshire A.. (2011). Dehydration affects brain structure and function in healthy adolescents. Hum. Brain Mapp. 32, 71–79. 10.1002/hbm.20999 20336685 PMC6869970

[B65] Kempton M. J. Ettinger U. Schmechtig A. Winter E. M. Smith L. Mcmorris T.. (2009). Effects of acute dehydration on brain morphology in healthy humans. Hum. Brain Mapp. 30, 291–298. 10.1002/hbm.20500 18064587 PMC6871128

[B66] Kharraz Y. Guerra J. Mann C. J. Serrano A. L. Muñoz-Cánoves P. (2013). Macrophage plasticity and the role of inflammation in skeletal muscle repair. Mediators Inflamm. 2013:491497. 10.1155/2013/491497 23509419 PMC3572642

[B67] Khowaja A. Choi I.-Y. Seaquist E. R. Öz G. (2014). *In vivo* Magnetic Resonance Spectroscopy of cerebral glycogen metabolism in animals and humans. Metab. Brain Dis. 30, 255–261. 10.1007/s11011-014-9530-7 24676563 PMC4392006

[B68] Kiyatkin E. A. Sharma H. S. (2009). Permeability of the blood–brain barrier depends on brain temperature. Neuroscience 161, 926–939. 10.1016/j.neuroscience.2009.04.004 19362131 PMC2694729

[B69] Knicker A. J. Renshaw I. Oldham A. R. Cairns S. P. (2011). Interactive processes link the multiple symptoms of fatigue in sport competition. Sports Med. 41, 307–328. 10.2165/11586070-000000000-00000 21425889

[B70] Kong J. Shepel P. N. Holden C. P. Mackiewicz M. Pack A. I. Geiger J. D. (2002). Brain glycogen decreases with increased periods of wakefulness: implications for homeostatic drive to sleep. J. Neurosci. 22, 5581–5587. 12097509 10.1523/JNEUROSCI.22-13-05581.2002 PMC6758203

[B71] Koslowsky M. Babkoff H. (1992). Meta-analysis of the relationship between total sleep deprivation and performance. Chronobiol. Int. 9, 132–136. 10.3109/07420529209064524 1533178

[B72] Lampropoulou S. I. Nowicky A. V. (2013). The effect of transcranial direct current stimulation on perception of effort in an isolated isometric elbow flexion task. Motor Control 17, 412–426. 24018733 10.1123/mcj.17.4.412

[B73] Leeder J. Glaister M. Pizzoferro K. Dawson J. Pedlar C. (2012). Sleep duration and quality in elite athletes measured using wristwatch actigraphy. J. Sports Sci. 30, 541–545. 10.1080/02640414.2012.660188 22329779

[B74] Lieberman H. R. Falco C. M. Slade S. S. (2002). Carbohydrate administration during a day of sustained aerobic activity improves vigilance, as assessed by a novel ambulatory monitoring device, and mood. Am. J. Clin. Nutr. 76, 120–127. 12081825 10.1093/ajcn/76.1.120

[B75] Lorist M. M. Tops M. (2003). Caffeine, fatigue, and cognition. Brain Cogn. 53, 82–94. 10.1016/S0278-2626(03)00206-9 14572506

[B76] Lui F. Colloca L. Duzzi D. Anchisi D. Benedetti F. Porro C. A. (2010). Neural bases of conditioned placebo analgesia. Pain 151, 816–824. 10.1016/j.pain.2010.09.021 20943318

[B77] Madsen K. Maclean D. A. Kiens B. Christensen D. (1996). Effects of glucose, glucose plus branched-chain amino acids, or placebo on bike performance over 100 km. J. Appl. Physiol. 81, 2644–2650. 9018517 10.1152/jappl.1996.81.6.2644

[B78] Mah C. D. Mah K. E. Kezirian E. J. Dement W. C. (2011). The effects of sleep extension on the athletic performance of collegiate basketball players. Sleep 34, 943. 10.5665/sleep.1132 21731144 PMC3119836

[B79] Marcora S. (2009). Perception of effort during exercise is independent of afferent feedback from skeletal muscles, heart, and lungs. J. Appl. Physiol. 106, 2060–2062. 10.1152/japplphysiol.90378.2008 18483166

[B80] Marcora S. M. (2008). Do we really need a central governor to explain brain regulation of exercise performance? Eur. J. Appl. Physiol. 104, 929–931. 10.1007/s00421-008-0818-3 18618133

[B81] Marcora S. M. Bosio A. De Morree H. M. (2008). Locomotor muscle fatigue increases cardiorespiratory responses and reduces performance during intense cycling exercise independently from metabolic stress. Am. J. Physiol. Regul. Integr. Comp. Physiol. 294, R874–R883. 10.1152/ajpregu.00678.2007 18184760

[B82] Marcora S. M. Staiano W. Manning V. (2009). Mental fatigue impairs physical performance in humans. J. Appl. Physiol. 106, 857–864. 10.1152/japplphysiol.91324.2008 19131473

[B83] Matsui T. Ishikawa T. Ito H. Okamoto M. Inoue K. Lee M. C.. (2012). Brain glycogen supercompensation following exhaustive exercise. J. Physiol. 590, 607–616. 10.1113/jphysiol.2011.217919 22063629 PMC3379704

[B84] Matsui T. Soya S. Okamoto M. Ichitani Y. Kawanaka K. Soya H. (2011). Brain glycogen decreases during prolonged exercise. J. Physiol. 589, 3383–3393. 10.1113/jphysiol.2010.203570 21521757 PMC3145946

[B85] Matsumoto K. Koba T. Hamada K. Sakurai M. Higuchi T. Miyata H. (2009). Branched-chain amino acid supplementation attenuates muscle soreness, muscle damage and inflammation during an intensive training program. J. Sports Med. Phys. Fitness 49, 424–431. 20087302

[B86] Meeusen R. Watson P. (2007). Amino acids and the brain: do they play a role in “central fatigue”? Int. J. Sport Nutr. Exerc. Metab. 17, s37–s46. 18577773 10.1123/ijsnem.17.s1.s37

[B87] Meeusen R. Watson P. Hasegawa H. Roelands B. Piacentini M. F. (2006). Central fatigue: the serotonin hypothesis and beyond. Sports Med. 36, 881–909. 10.2165/00007256-200636100-00006 17004850

[B88] Meijman T. F. (2000). The theory of the stop-emotion: On the functionality of fatigue, in Ergonomics and Safety for Global Business Quality and Production, eds Pogorski D. Karwowski W. (Warschaw: CIOP), 45–50.

[B89] Michalsen A. Grossman P. Lehmann N. Knoblauch N. T. Paul A. Moebus S.. (2005). Psychological and quality-of-life outcomes from a comprehensive stress reduction and lifestyle program in patients with coronary artery disease: results of a randomized trial. Psychother. Psychosom. 74, 344–352. 10.1159/000087781 16244510

[B90] Minett G. M. Duffield R. (2014). Is recovery driven by central or peripheral factors? a role for the brain in recovery following intermittent-sprint exercise. Front. Physiol. 5:24. 10.3389/fphys.2014.00024 24550837 PMC3909945

[B91] Minett G. M. Duffield R. Billaut F. Cannon J. Portus M. Marino F. E. (2013). Cold-water immersion decreases cerebral oxygenation but improves recovery after intermittent−sprint exercise in the heat. Scand. J. Med. Sci. Sports 24, 656–666. 10.1111/sms.12060 23458430

[B92] Minett G. M. Duffield R. Kellett A. Portus M. (2012). Effects of mixed-method cooling on recovery of medium-fast bowling performance in hot conditions on consecutive days. J. Sports Sci. 30, 1387–1396. 10.1080/02640414.2012.709267 22867101

[B93] Mittleman K. D. Ricci M. R. Bailey S. P. (1998). Branched-chain amino acids prolong exercise during heat stress in men and women. Med. Sci. Sports Exerc. 30, 83. 10.1097/00005768-199801000-00012 9475648

[B94] Morrison S. A. Cheung S. S. Hurst R. D. Cotter J. D. (2013). Cognitive function and blood-brain barrier permeability during exercise in the heat: effect of fitness and bovine colostrum supplementation. J. Therm. Biol. 38, 374–383 10.1016/j.jtherbio.2013.05.002

[B95] Mosso A. (1914). Fatigue. Transl. by Drummond M. Drummond W. G. London: Allen & Unwin).

[B96] Murphy P. J. Campbell S. (1997). Nighttime drop in body temperature: a physiological trigger for sleep onset? Sleep 20, 505–511. 9322266 10.1093/sleep/20.7.505

[B97] Nehlsen-Cannarella S. Fagoaga O. Nieman D. Henson D. Butterworth D. Schmitt R.. (1997). Carbohydrate and the cytokine response to 2.5 h of running. J. Appl. Physiol. 82, 1662–1667. 9134917 10.1152/jappl.1997.82.5.1662

[B98] Neill D. B. Justice J. B. (1981). An hypothesis for a behavioral function of dopaminergic transmission in nucleus accumbens, in The Neurobiology of the Nucleus Accumbens, eds Chronister R. B. Defrance J. F. (Brunswick: Haer Institute for Electrophysiological Research), 343–350.

[B99] Nielsen B. Hyldig T. Bidstrup F. Gonzalez-Alonso J. Christoffersen G. (2001). Brain activity and fatigue during prolonged exercise in the heat. Pflügers Arch. 442, 41–48. 10.1007/s004240100515 11374067

[B100] Nieman D. C. Nehlsen-Cannarella S. L. Fagoaga O. R. Henson D. Utter A. Davis J. M.. (1998). Influence of mode and carbohydrate on the cytokine response to heavy exertion. Med. Sci. Sports Exerc. 30, 671–678. 10.1097/00005768-199805000-00005 9588607

[B101] Nierwińska K. Malecka E. Chalimoniuk M. Żebrowska A. Langfort J. (2008). Blood-brain barrier and exercise – a short review. J. Hum. Kinet. 19, 83–92 10.2478/v10078-008-0006-x

[B102] Nybo L. (2010). CNS fatigue provoked by prolonged exercise in the heat. Front. Biosci. (Elite. Ed). 2, 779–792. 10.2741/e138 20036922

[B103] Nybo L. Dalsgaard M. K. Steensberg A. Møller K. Secher N. H. (2005). Cerebral ammonia uptake and accumulation during prolonged exercise in humans. J. Physiol. 563, 285–290. 10.1113/jphysiol.2004.075838 15611036 PMC1665558

[B104] Nybo L. Møller K. Volianitis S. Nielsen B. Secher N. H. (2002a). Effects of hyperthermia on cerebral blood flow and metabolism during prolonged exercise in humans. J. Appl. Physiol. 93, 58–64. 10.1152/japplphysiol.00049.2002 12070186

[B105] Nybo L. Nielsen B. Blomstrand E. Møller K. Secher N. (2003). Neurohumoral responses during prolonged exercise in humans. J. Appl. Physiol. 95, 1125–1131. 10.1152/japplphysiol.00241.2003 12754171

[B106] Nybo L. Secher N. H. Nielsen B. (2002b). Inadequate heat release from the human brain during prolonged exercise with hyperthermia. J. Physiol. 545, 697–704. 10.1113/jphysiol.2002.030023 12456844 PMC2290690

[B107] Nybo L. Wanscher M. Secher N. H. (2014). Influence of intranasal and carotid cooling on cerebral temperature balance and oxygenation. Front. Physiol. 1:79. 10.3389/fphys.2014.00079 24578693 PMC3936139

[B108] Okano A. H. Fontes E. B. Montenegro R. A. Farinatti P. D. T. V. Cyrino E. S. Li L. M.. (2013). Brain stimulation modulates the autonomic nervous system, rating of perceived exertion and performance during maximal exercise. Br. J. Sports Med. [Epub ahead of print]. 10.1136/bjsports-2012-091658 23446641

[B109] Pageaux B. Lepers R. Dietz K. C. Marcora S. M. (2014). Response inhibition impairs subsequent self-paced endurance performance. Eur. J. Appl. Physiol. 114, 1095–1105. 10.1007/s00421-014-2838-5 24531591

[B110] Pageaux B. Marcora S. Lepers L. (2013). Prolonged mental exertion does not alter neuromuscular function of the knee extensors. Med. Sci. Sports Exerc. 45, 2254–2264. 10.1249/MSS.0b013e31829b504a 23698244

[B111] Parkin J. Carey M. Zhao S. Febbraio M. (1999). Effect of ambient temperature on human skeletal muscle metabolism during fatiguing submaximal exercise. J. Appl. Physiol. 86, 902–908. 10066703 10.1152/jappl.1999.86.3.902

[B112] Parks R. W. Loewenstein D. A. Dodrill K. L. Barker W. W. Yoshii F. Chang J. Y.. (1988). Cerebral metabolic effects of a verbal fluency test: a PET scan study. J. Clin. Exp. Neuropsychol. 10, 565–575. 10.1080/01688638808402795 3265709

[B113] Patrick G. Gilbert J. A. (1896). Studies from the psychological laboratory of the University of Iowa: on the effects of loss of sleep. Psychol. Rev. 3, 469 10.1037/h0075739

[B114] Paus T. Koski L. Caramanos Z. Westbury C. (1998). Regional differences in the effects of task difficulty and motor output on blood flow response in the human anterior cingulate cortex: a review of 107 PET activation studies. Neuroreport 9, R37–R47. 10.1097/00001756-199806220-00001 9674567

[B115] Pilcher J. J. Huffcutt A. J. (1996). Effects of sleep deprivation on performance: a meta-analysis. Sleep 19, 318–326. 8776790 10.1093/sleep/19.4.318

[B116] Pointon M. Duffield R. Cannon J. Marino F. E. (2012). Cold water immersion recovery following intermittent-sprint exercise in the heat. Eur. J. Appl. Physiol. 112, 2483–2494. 10.1007/s00421-011-2218-3 22057508

[B117] Portas C. M. Rees G. Howseman A. Josephs O. Turner R. Frith C. D. (1998). A specific role for the thalamus in mediating the interaction of attention and arousal in humans. J. Neurosci. 18, 8979–8989. 9787003 10.1523/JNEUROSCI.18-21-08979.1998 PMC6793555

[B118] Pyne D. Gleeson M. Mcdonald W. Clancy R. Perry C. Fricker P. (2000). Training strategies to maintain immunocompetence in athletes. Int. J. Sports Med. 21, S51. 10.1055/s-2000-1452 10893025

[B119] Raymann R. J. Swaab D. F. Van Someren E. J. (2008). Skin deep: enhanced sleep depth by cutaneous temperature manipulation. Brain 131, 500–513. 10.1093/brain/awm315 18192289

[B120] Reis P. M. Hebenstreit F. Gabsteiger F. Von Tscharner V. Lochmann M. (2014). Methodological aspects of EEG and body dynamics measurements during motion. Front. Hum. Neurosci. 8:156. 10.3389/fnhum.2014.00156 24715858 PMC3970018

[B121] Robey E. Dawson B. Halson S. Goodman C. Gregson W. Eastwood P. (2013a). Post-exercise cold water immersion: effect on core temperature and melatonin responses. Eur. J. Appl. Physiol. 113, 305–311. 10.1007/s00421-012-2436-3 22706550

[B122] Robey E. Dawson B. Halson S. Gregson W. King S. Goodman C.. (2013b). Effect of evening postexercise cold water immersion on subsequent sleep. Med. Sci. Sports Exerc. 45, 1394–1402. 10.1249/MSS.0b013e318287f321 23377833

[B123] Robson-Ansley P. J. Milander L. D. Collins M. Noakes T. D. (2004). Acute interleukin-6 administration impairs athletic performance in healthy, trained male runners. Can. J. Appl. Physiol. 29, 411–418. 10.1139/h04-026 15317982

[B124] Roelands B. Hasegawa H. Watson P. Piacentini M. Buyse L. De Schutter G.. (2008). The effects of acute dopamine reuptake inhibition on performance. Med. Sci. Sports Exerc. 40, 879–885. 10.1249/MSS.0b013e3181659c4d 18408610

[B125] Rollo I. Williams C. (2010). Influence of ingesting a carbohydrate-electrolyte solution before and during a 1-hour run in fed endurance-trained runners. J. Sports Sci. 28, 593–601. 10.1080/02640410903582784 20391081

[B126] Rudebeck P. H. Walton M. E. Smyth A. N. Bannerman D. M. Rushworth M. F. (2006). Separate neural pathways process different decision costs. Nat. Neurosci. 9, 1161–1168. 10.1038/nn1756 16921368

[B127] Ruh,é H. G. Mason N. S. Schene A. H. (2007). Mood is indirectly related to serotonin, norepinephrine and dopamine levels in humans: a meta-analysis of monoamine depletion studies. Mol. Psychiatry 12, 331–359. 10.1038/sj.mp.4001949 17389902

[B128] Salamone J. D. Aberman J. E. Sokolowski J. D. Cousins M. S. (1999). Nucleus accumbens dopamine and rate of responding: neurochemical and behavioral studies. Psychobiology 27, 236–247. 16001115

[B129] Secher N. H. Seifert T. Van Lieshout J. J. (2008). Cerebral blood flow and metabolism during exercise: implications for fatigue. J. Appl. Physiol. 104, 306–314. 10.1152/japplphysiol.00853.2007 17962575

[B130] Sinnerton S. Reilly T. (1992). Effects of sleep loss and time of day in swimmers, in Biomechanics and Medicine in Swimming: Swimming Science IV, eds Maclaren D. Reilly T. Lees A. (London: E and F.N. Spon), 399–405.

[B131] Smirmaul B. P. C. Dantas J. L. Nakamura F. Y. Pereira G. (2013). The psychobiological model: a new explanation to intensity regulation and (in) tolerance in endurance exercise. Revista Brasileira de Educação Física e Esporte 27, 333–340 10.1590/S1807-55092013005000008

[B132] Smriga M. Kameishi M. Tanaka T. Kondoh T. Torii K. (2002). Preference for a solution of branched-chain amino acids plus glutamine and arginine correlates with free running activity in rats: involvement of serotonergic-dependent processes of lateral hypothalamus. Nutr. Neurosci. 5, 189–199. 10.1080/10284150290028936 12041875

[B133] Stanley J. Leveritt M. Peake J. M. (2010). Thermoregulatory responses to ice-slush beverage ingestion and exercise in the heat. Eur. J. Appl. Physiol. 110, 1163–1173. 10.1007/s00421-010-1607-3 20714767

[B134] Stone M. Thomas K. Wilkinson M. Jones A. St Clair G. A. Thompson K. (2012). Effects of deception on exercise performance: implications for determinants of fatigue in humans. Med. Sci. Sports Exerc. 44, 534–541. 10.1249/MSS.0b013e318232cf77 21886012

[B135] Streitbürger D.-P. Möller H. E. Tittgemeyer M. Hund-Georgiadis M. Schroeter M. L. Mueller K. (2012). Investigating structural brain changes of dehydration using voxel-based morphometry. PLoS ONE 7:e44195. 10.1371/journal.pone.0044195 22952926 PMC3430653

[B136] Strüder H. Hollmann W. Platen P. Donike M. Gotzmann A. Weber K. (1998). Influence of paroxetine, branched-chain amino acids and tyrosine on neuroendocrine system responses and fatigue in humans. Horm. Metab. Res. 30, 188–194. 10.1055/s-2007-978864 9623632

[B137] Strüder H. Weicker H. (2001a). Physiology and pathophysiology of the serotonergic system and its implications on mental and physical performance. Part I. Int. J. Sports Med. 22, 467–481. 10.1055/s-2001-17605 11590474

[B138] Strüder H. Weicker H. (2001b). Physiology and pathophysiology of the serotonergic system and its implications on mental and physical performance. Part II. Int. J. Sports Med. 22, 482–497. 10.1055/s-2001-17606 11590475

[B139] Swart J. (2009). Exercising with reserve: evidence that the central nervous system regulates prolonged exercise performance. Br. J. Sports Med. 43, 782–788. 10.1136/bjsm.2008.055889 19052141

[B140] Szechtman H. Talangbayan H. Ganaran G. Dai H. Eilam D. (1994). Dynamics of behavioral sensitization induced by the dopamine agonist quinpirole and a proposed central energy control mechanism. Psychopharmacology 115, 95–104. 10.1007/BF02244757 7862919

[B141] Teng E. Lastella M. Roach G. Sargent C. (2011). The effect of training load on sleep quality and sleep perception in elite male cyclists, in Little Clock, Big Clock: Molecular to Physiological Clocks, eds Kennedy G. Sargent C. (Melbourne: Australasian Chronobiology Society, Victoria University), 5–10.

[B142] Thomas M. Sing H. Belenky G. Holcomb H. Mayberg H. Dannals R.. (2000). Neural basis of alertness and cognitive performance impairments during sleepiness. I. Effects of 24 h of sleep deprivation on waking human regional brain activity. J. Sleep Res. 9, 335–352. 10.1046/j.1365-2869.2000.00225.x 11123521

[B143] Tipton K. D. Rasmussen B. B. Miller S. L. Wolf S. E. Owens-Stovall S. K. Petrini B. E.. (2001). Timing of amino acid-carbohydrate ingestion alters anabolic response of muscle to resistance exercise. Am. J. Physiol. Endocrinol. Metab. 281, E197–E206. 11440894 10.1152/ajpendo.2001.281.2.E197

[B144] Trangmar S. J. Chiesa S. T. Stock C. G. Kalsi K. K. Secher N. H. González-Alonso J. (2014). Dehydration affects cerebral blood flow but not its metabolic rate for oxygen during maximal exercise in trained humans. J. Physiol. 592, 3143–3160. 10.1113/jphysiol.2014.272104 24835170 PMC4214665

[B145] Tumilty L. Davison G. Beckmann M. Thatcher R. (2013). Acute oral administration of a tyrosine and phenylalanine-free amino acid mixture reduces exercise capacity in the heat. Eur. J. Appl. Physiol. 113, 1511–1522. 10.1007/s00421-012-2577-4 23288035

[B146] Vaile J. Halson S. Gill N. Dawson B. (2008a). Effect of cold water immersion on repeat cycling performance and thermoregulation in the heat. J. Sports Sci. 26, 431–440. 10.1080/02640410701567425 18274940

[B147] Vaile J. Halson S. Gill N. Dawson B. (2008b). Effect of hydrotherapy on the signs and symptoms of delayed onset muscle soreness. Eur. J. Appl. Physiol. 102, 447–455. 10.1007/s00421-007-0605-6 17978833

[B148] Van Hall G. Raaymakers J. Saris W. H. M. Wagenmakers A. J. M. (1995). Ingestion of branched-chain amino acids and tryptophan during sustained exercise in man: failure to affect performance. J. Physiol. 486, 789–794. 10.1113/jphysiol.1995.sp020854 7473239 PMC1156566

[B149] Varnier M. Sarto P. Martines D. Lora L. Carmignoto F. Leese G. P.. (1994). Effect of infusing branched-chain amino acid during incremental exercise with reduced muscle glycogen content. Eur. J. Appl. Physiol. Occup. Physiol. 69, 26–31. 10.1007/BF00867923 7957152

[B150] Waller A. D. (1891). The sense of effort: an objective study. Brain 14, 179–249 10.1093/brain/14.2-3.179

[B151] Walton M. E. Bannerman D. M. Alterescu K. Rushworth M. F. (2003). Functional specialization within medial frontal cortex of the anterior cingulate for evaluating effort-related decisions. J. Neurosci. 23, 6475–6479. 12878688 10.1523/JNEUROSCI.23-16-06475.2003 PMC6740644

[B152] Walton M. Kennerley S. Bannerman D. Phillips P. Rushworth M. F. (2006). Weighing up the benefits of work: behavioral and neural analyses of effort-related decision making. Neural Netw. 19, 1302–1314. 10.1016/j.neunet.2006.03.005 16949252 PMC2519033

[B153] Watson A. El-Deredy W. Iannetti G. D. Lloyd D. Tracey I. Vogt B. A.. (2009). Placebo conditioning and placebo analgesia modulate a common brain network during pain anticipation and perception. Pain 145, 24–30. 10.1016/j.pain.2009.04.003 19523766 PMC2743811

[B154] Watson P. Black K. E. Clark S. C. Maughan R. J. (2006). Exercise in the heat: effect of fluid ingestion on blood-brain barrier permeability. Med. Sci. Sports Exerc. 38, 2118–2124. 10.1249/01.mss.0000235356.31932.0a 17146318

[B155] Watson P. Head K. Pitiot A. Morris P. Maughan R. J. (2010). Effect of exercise and heat-induced hypohydration on brain volume. Med. Sci. Sports Exerc. 42, 2197–2204. 10.1249/MSS.0b013e3181e39788 20421835

[B156] Watson P. Shirreffs S. M. Maughan R. J. (2005). Blood-brain barrier integrity may be threatened by exercise in a warm environment. Am. J. Physiol. Regul. Integ. Comp. Physiol. 288, R1689–R1694. 10.1152/ajpregu.00676.2004 15650123

[B157] Wilkinson D. J. Smeeton N. J. Watt P. W. (2010). Ammonia metabolism, the brain and fatigue; revisiting the link. Progr. Neurobiol. 91, 200–219. 10.1016/j.pneurobio.2010.01.012 20138956

[B158] Williamson J. Mccoll R. Mathews D. Mitchell J. Raven P. Morgan W. (2002). Brain activation by central command during actual and imagined handgrip under hypnosis. J. Appl. Physiol. 92, 1317–1324. 10.1152/japplphysiol.00939.2001 11842073

[B159] Williamson J. W. Fadel P. J. Mitchell J. H. (2006). New insights into central cardiovascular control during exercise in humans: a central command update. Exp. Physiol. 91, 51–58. 10.1113/expphysiol.2005.032037 16239250

[B160] Williamson J. W. Mccoll R. Mathews D. Mitchell J. H. Raven P. B. Morgans W. P. (2001). Hypnotic manipulation of effort sense during dynamic exercise: cardiovascular responses and brain activation. J. Appl. Physiol. 90, 1392–1399. 11247939 10.1152/jappl.2001.90.4.1392

[B161] Winchester R. Turer L. A. Thomas K. Ansley L. Thompson K. G. Micklewright D. (2012). Observer effects on the rating of percieved exertion and affect during exercise in recreationally active males. Percept. Mot. Skills 115, 213–227. 10.2466/25.07.05.PMS.115.4.213-227 23033758

[B162] Wright R. A. (1998). Ability perception and cardiovascular response to behavioral challenge, in Personal Control in Action: Cognitive and Motivational Mechanisms, eds Kofka M. Weary G. Sedek G. (New York, NY: Guilford), 197–232.

[B163] Wu J. C. Gillin J. Buchsbaum M. S. Hershey T. (1991). The effect of sleep deprivation on cerebral glucose metabolic rate in normal humans assessed with positron emission tomography. Sleep 14, 155–162. 1866529

[B164] Xie L. Kang H. Xu Q. Chen M. J. Liao Y. Thiyagarajan M.. (2013). Sleep drives metabolite clearance from the adult brain. Science 342, 373–377. 10.1126/science.1241224 24136970 PMC3880190

